# Saliva gas analysis method for non-invasive detection of diseased organ

**DOI:** 10.1016/j.mex.2026.104210

**Published:** 2026-10-15

**Authors:** Vicky Nalawade, Vinayak K. Bairagi

**Affiliations:** a Research Scholar, Electronics and Telecommunication Engineering, AISSMS Institute of Information Technology, Savitribai Phule Pune University, Pune, Maharashtra, 411007, India; b Dean Research and Development, Professor, Electronics and Telecommunication Engineering, AISSMS Institute of Information Technology, Savitribai Phule Pune University, Pune, Maharashtra, 411007, India; c Assistant Professor, Department of Electronics and Telecommunication Engineering, Kasegaon Education Society’s Rajarambapu Institute of Technology, Affiliated to Shivaji University, Sakharale, Maharashtra, 415414, India

**Keywords:** Saliva gas analysis, Disease detection, Conductivity, Methane, Ammonia, Oxygen

## Abstract

Saliva contains volatile organic compounds (VOCs) that reflect metabolic activity and can be indicative of underlying health conditions. Saliva contains a variety of biomarkers, including DNA, RNA, proteins, enzymes, hormones and metabolites, making it a valuable and non-invasive sample for medical diagnostics. The dis-functioning of liver, kidney, digestion system (stomach) and oral health results in leaving traces of VOCs in saliva. This is often refereed as mouth odour or halitosis. This paper discusses a non-invasive method to identify diseased organ from saliva gas analysis. An electronic sensor array comprising of sensors for sensing conductivity, oxygen, methane and ammonia gas is fabricated for conducting analysis of gases evolving from saliva sample. The quantity of these gases is compared with a predetermined threshold to identify the diseased organ. This analysis is compared with the pathology lab report for validation purpose.

• Being non-invasive it avoids discomfort of blood draws, making it ideal for continuous monitoring.
• Salivary gas bypasses respiratory variables of traditional breath analyser tests.
• This method assist physicians for by and large diagnosis of diseased organ.

## Graphical abstract

**Figure:**
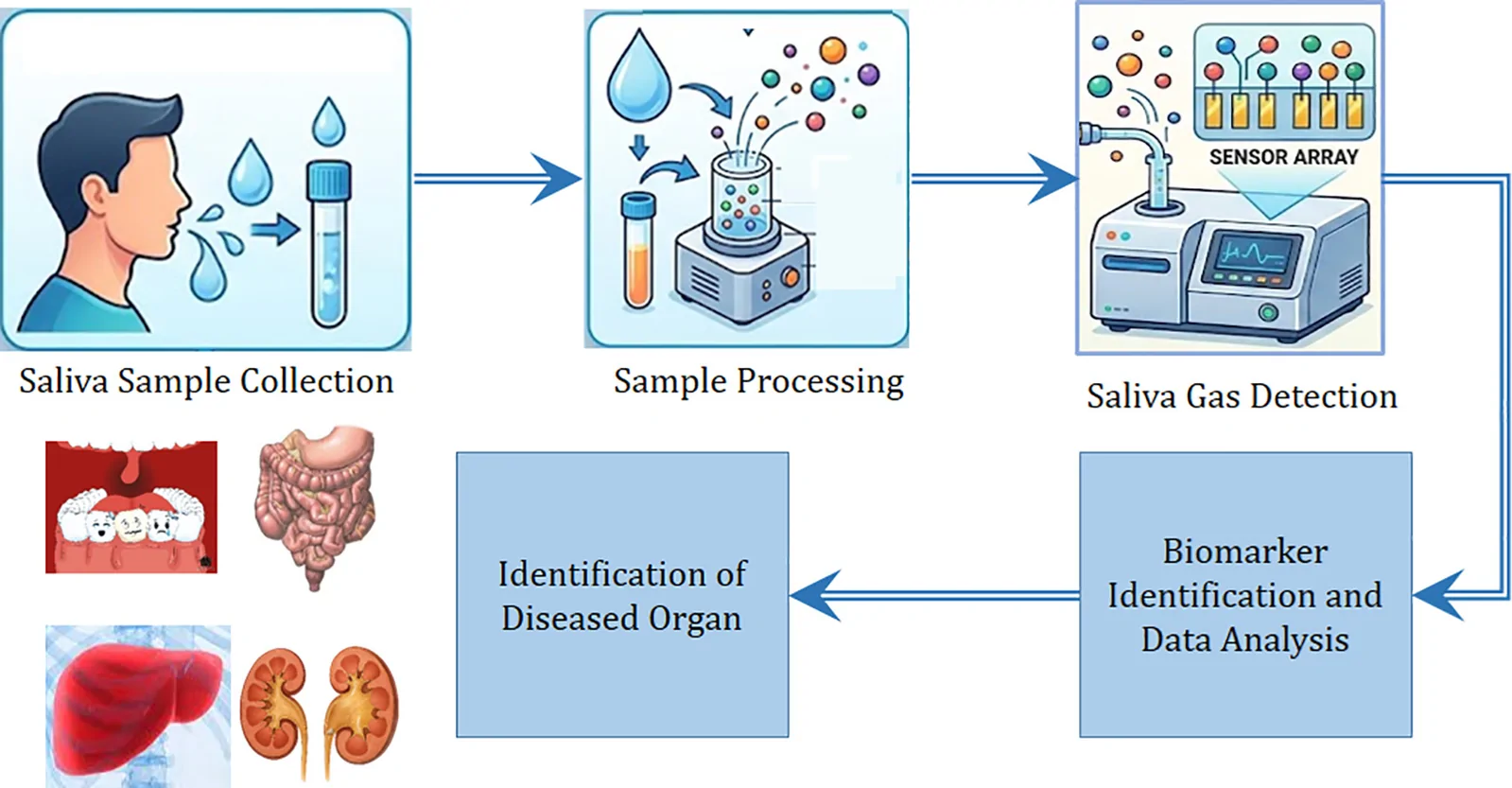

## Specifications table

**Table:** 

**Subject area**	Bioinformatics
**More specific subject area**	*Saliva*
**Name of your method**	*Saliva gas analysis with sensor array*
**Name and reference of original method**	*—*
**Resource availability**	*Saliva collection via: Sterile Test Tube* *Inspection Chamber Sensors:* *Oxygen – ASAIR AO-03* *Methane - TGS6812-D00* *Ammonia - Dfrobot Fermion MEMS NH3* *Conductivity - LFS 1107* *Ambient Temperature - DHT11* *Data Collection and Processor: Arduino Uno* *Display: 16* *×* *2 LCD*

## Background

Saliva gas analysis is an emerging non-invasive tool for detecting diseases by identifying VOCs and gases in saliva that reflect systemic and localized health conditions. These biomarkers, provide critical insights into metabolic, inflammatory, and pathological processes. Saliva’s rich composition encompassing hormones, enzymes, DNA, RNA, proteins, metabolites, and microbial content enables diverse diagnostic approaches [1]. Biochemical analysis measure cortisol for stress assessment or amylase for pancreatic health, while genomic analyses detect mutations or viral pathogens via PCR. Proteomic studies identify disease-specific proteins [2]. Metabolomics and gas analysis focus on VOCs, such as acetone for diabetes monitoring or hydrogen sulfide for periodontal disease, using advanced tools like gas chromatography. Saliva also serves as a window to the oral microbiome, revealing dysbiosis associated with periodontitis or systemic illnesses, and facilitates viral load tracking for HIV or hepatitis [3]. Innovations in point-of-care devices, including smartphone-integrated sensors and lateral flow assays allow rapid, on-site testing for infections like COVID-19 or drug screening [4]. Saliva-based biomarker identification is an innovative, non-invasive alternative to traditional diagnostic methods, offering a painless and convenient approach for early diagnostic disease and health monitoring [5]. The integration of advanced techniques, such as nanotechnology, artificial intelligence, and biosensors, has significantly improved the sensitivity and specificity of biomarker detection, enabling precise and real-time diagnostics.

The VOC composition of saliva is a good indicator of abdominal and lung health. The static stir bar extraction technique [6] with gas chromatography demonstrated high sensitivity and precision as an analytical method for detecting a diverse array of small organic molecules in saliva, effectively accommodating compounds with varying functional groups. Similar to the arterial blood gas analysis, saliva gas analysis [7] in clinical environment can reduce the logistic burden of critically ill patients. The saliva collection method can be a source of contamination and hence simple methods are adopted for saliva collection [8]. Saliva collection methods are designed to ensure sample integrity while accommodating diverse clinical and research needs. Unstimulated saliva is typically collected by allowing saliva to passively accumulate in the mouth before expectorating into a sterile tube, ideal for measuring baseline biomarkers like cortisol [9]. Stimulated saliva, induced by chewing inert materials or applying citric acid to the tongue, enhances flow rate and volume, useful for analyzing electrolytes or enzymes. Specialized tools like absorbent swabs or oral wash techniques, simplify collection in vulnerable populations [10].

The salivary glands are highly vascularized organs supplied by an extensive capillary network. Small, lipophilic molecules and systemic VOCs circulating in the bloodstream partition rapidly across the thin capillary-alveolar-like barrier into the saliva matrix via passive diffusion. Consequently, saliva acts as a biochemical mirror of blood plasma. By analyzing the gas phase of saliva rather than the complex liquid matrix itself, researchers can capture these systemic biomarkers while bypassing the protein-heavy, viscous interferences that typically complicate liquid saliva assays. This paper introduces saliva gas analysis method to detection of diseased organs with electronic sensors array. This method provides a reproducible and highly sensitive framework for identifying distinct organ-specific volatile profiles.

## Method details

In the detection of diseased organ with saliva gas analysis, at first saliva sample is obtained from the individual. The Fig. 1 illustrates the experimental procedure. The consideration and preparations in each stage are listed below-

1. Pre collection advise / Guideline
  a. Avoid eating, smoking, drinking or chewing gum for 60 min before sample collection
  b. Avoid brushing teeth or using mouthwash just prior to sample collection
2. Saliva Sample Collection
  a. Sterile saliva collection tube / vial
  b. Provide cotton pad to the person whose saliva sample is to be collected
  c. Chew the cotton pad until it gets sufficiently wet
  d. Spit the cotton pad
  e. Squeeze the cotton pad and collect the saliva in test tube
  f. The collected saliva quantity shall be 2 – 5ml
  g. If saliva quantity is less, above procedure shall be repeated.
3. Data creation
  a. Place 2 ml sample in the examination chamber
  b. Read the data of conductivity, oxygen, ammonia and methane sensors.
  c. Records sensor data and mark with appropriate labels
4. Pathological data
  a. The pathological data may be used to correlate the disease data with the evolved gas data
  b. Clinical expertise shall be availed for disease diagnosis
5. Data validation
  a. The evolved gases recorded during dataset creation are compared with thresholds for detecting diseases in human
  b. To validate the disease detection, the pathological data is used.

**Fig. 1:**
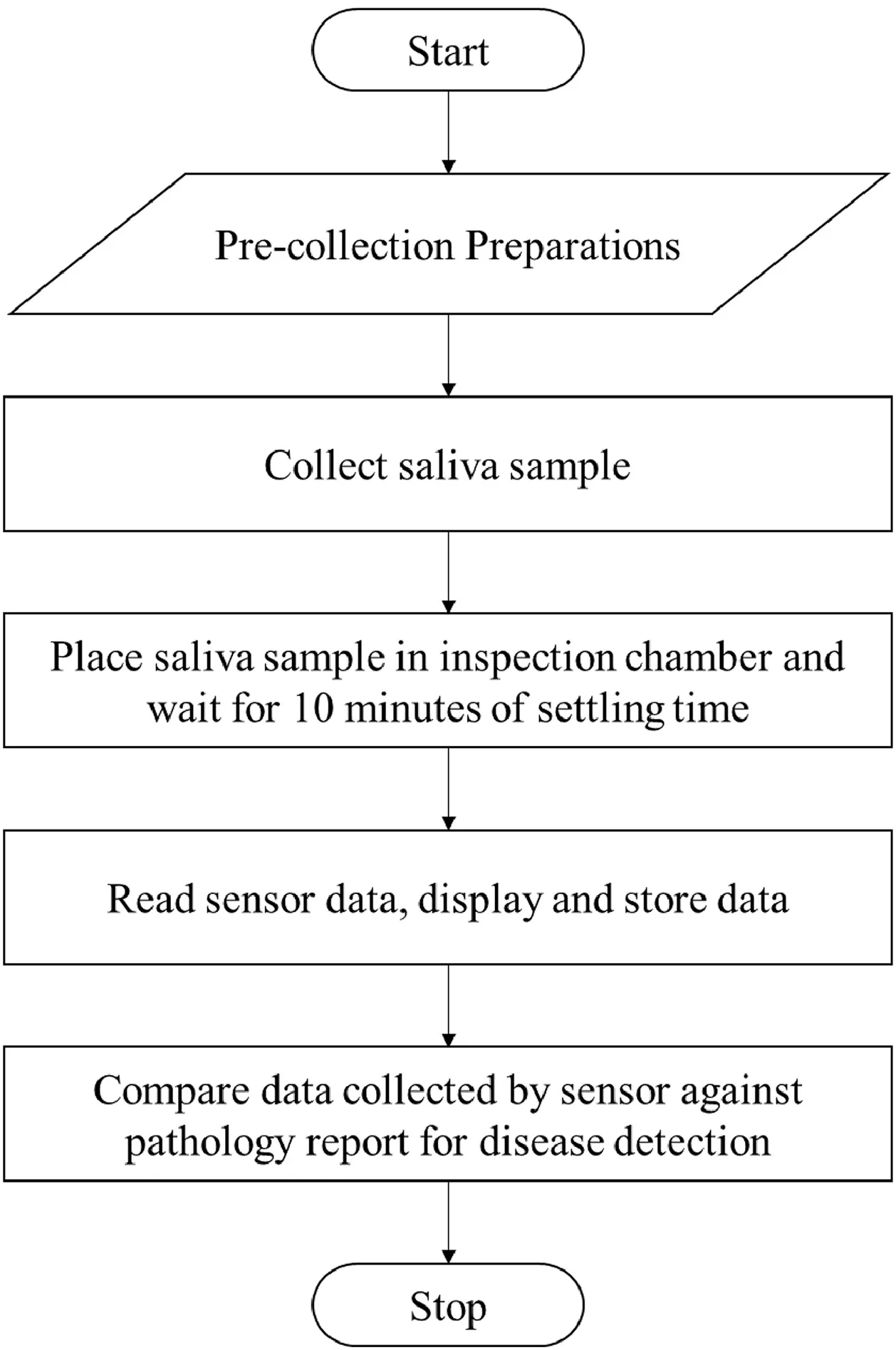
Experimentation work flow.

The collected saliva sample is transferred to a watch glass and placed in the inspection chamber. The inspection chamber is constructed with glass material in dome shape. The gas sensor array is placed at the top of the inspection chamber for monitoring oxygen, methane and ammonia. At the floor of the inspection chamber a provision is made to place a watch glass containing saliva sample. The watch glass is assembled with conductivity sensor and inspection chamber is equipped with sensors for measuring oxygen, methane and ammonia gases. A dedicated experimental setup for saliva gas sensing is constructed by using conductivity sensor (LFS 1107), methane sensor (TGS6812-D00), Ammonia sensor (Dfrobot Fermion MEMS NH3), and oxygen sensor (ASAIR AO-03). The data collected by these sensors is processed by Arduino Uno and recorded displayed on a 16 × 2 LCD display. The data of all these sensors is collected by a controller and displayed on LCD display. The data is marked and compared with thresholds for detection of diseased organ (liver / kidney/ oral infection). This data is also compared with pathological report and post analysis by clinical experts. The experimental setup is seen in Fig. 2. For detection of diseased organ, the sensor reading is compared with threshold levels as mentioned in Table 1. Fig. 3 shows the details of the inspection chamber and sensor placement.

**Fig. 2:**
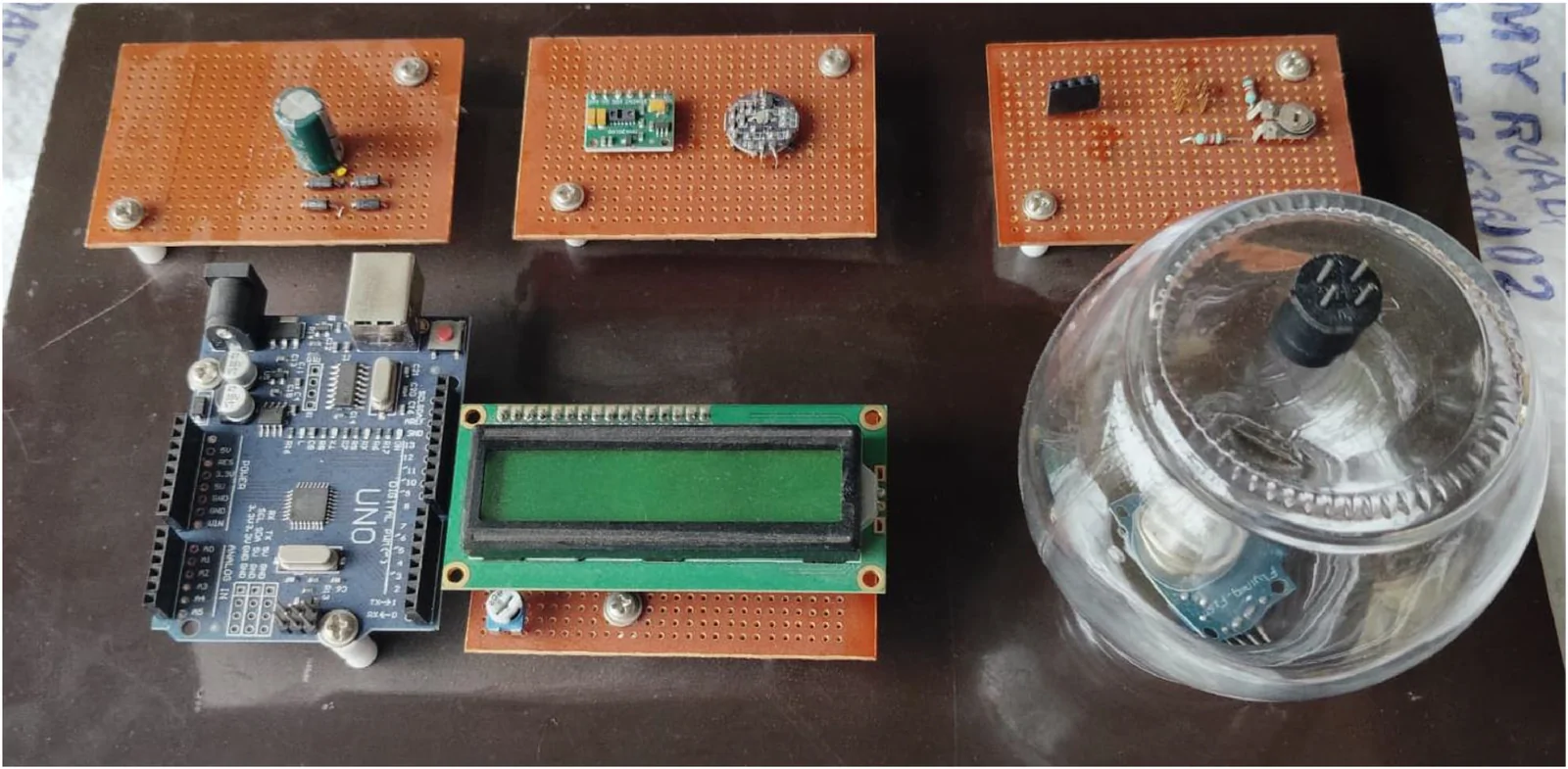
Experimental Setup.

**Table 1:** Saliva Gas parameters sensors, normal range and illness range.

Gas / Parameter	Normal value range	Illness range	Disease Type
Reactive Oxygen	260 – 300	< 250	Oral decay
Methane	0 – 150	> 150	Gastrointestinal disorder Liver functioning
Ammonia	0 – 95	> 95	Kidney functioning Creatinine level
Conductivity	3400 – 3600	< 3400 & >3600	Digestion, Kidney disease if conductivity > 3600

**Fig. 3:**
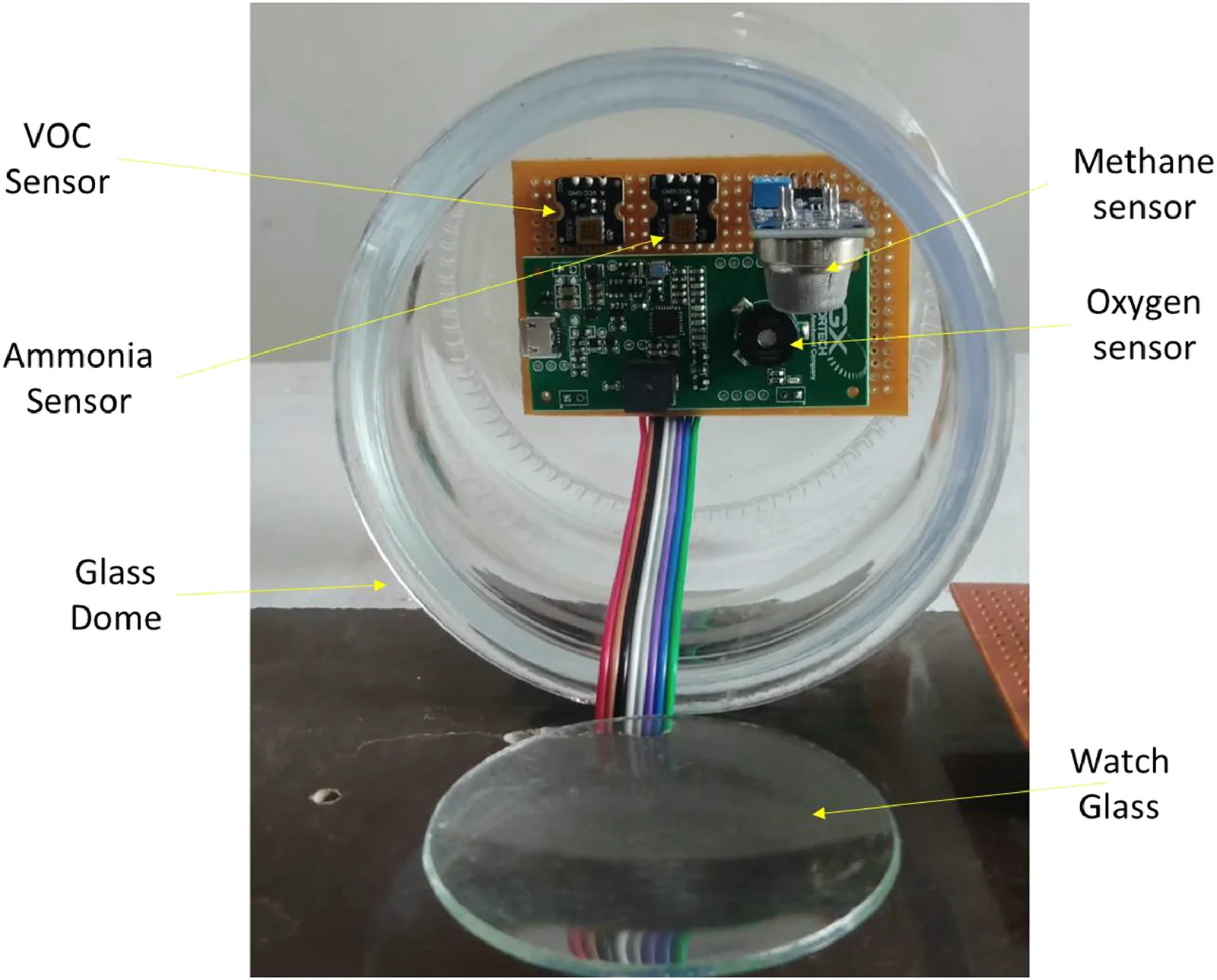
Photograph of inspection chamber sensor arrangements.

The saliva sample is taken in the quantity of 2 to 3 ml. A part of collected sample (0.3 - 0.5 ml) and is placed on conductivity sensor for measurement of saliva conductivity. The conductivity sensor assist in diagnosis of periodontal disease (dental health) and kidney functioning. The remaining quantity of saliva is taken into a watch glass and placed in inspection chamber. This sample spends a warm-up time of 15 min inside the inspection chamber. The inspection chamber is constructed in such a way that it does not allows the evaporated gases from saliva to escape. The evolved gases from saliva are sensed by various gas sensors in the inspection chamber. The data of sensors is processed through a controller board which is programmed to compare the incoming sensor data with threshold levels for diagnosis and diseased organ detection. The observation results for various samples of saliva with disease detection are mentioned in Table 2. The disease detection is also compared with pathological reports for validation. It is seen that the disease detection with saliva gas analysis closely matches pathological testing with blood test, urine test and stool test results for iprimary screening of diseased organ.

**Table 2:** Disease detection from saliva gas analysis and comparison with lab diagnosis.

ID No.	Conductivity (mS/cm)	Oxygen (ADC data)	Methane (ADC data)	Ammonia (ADC data)	Disease (Gas analysis)	Pathological Lab report	Diagnosis
Saliva_01	3450	263	124	81	—	—	Healthy
Saliva_02	3478	268	101	87	—	—	Healthy
Saliva_03	3677	274	121	99	Kidney	Creatinine – 1.43	Urine infection
Saliva_04	3490	278	118	81	—	—	Healthy
Saliva_05	3392	230	259	117	Stomach	Stool test Bacteria positive Creatinine – 1.37	Loose motion (Gastro)
Saliva_06	3352	265	131	102	Stomach	Stool test Bacteria positive Urine test: amber colour	Gastro
Saliva_07	3512	268	153	86	Liver	Liver functioning test ALT – 69 AST - 59	Stomach ache
Saliva_08	3524	264	201	81	Liver	Liver functioning test ALT – 67 AST – 56	Stomach ache
Saliva_09	3465	235	157	118	Kidney	Creatinine – 1.56	Urine infection
Saliva_10	3511	269	145	99	Stomach	Stool test Bacterial infection	Stomach ache

## Method validation

The resutls of saliva gas analysis method for detection of diseased organ are compared to the pathology lab report for validation. A sample pathilogy report of saliva sample “Saliva_03″ is shown in Fig. 4. The pathology report was taken on blood and urine test as the patient suffered fromfever and weakness. The pathology report shown serum creatinine level of 1.43 which revealed the diagnosis of urine infection i.e. kidney as diseased organ.For every saliva sample the pathology report validation is conducted. For pathology report, samples of blood, urine or stool are taken as applicatble for the individual case.

**Fig. 4:**
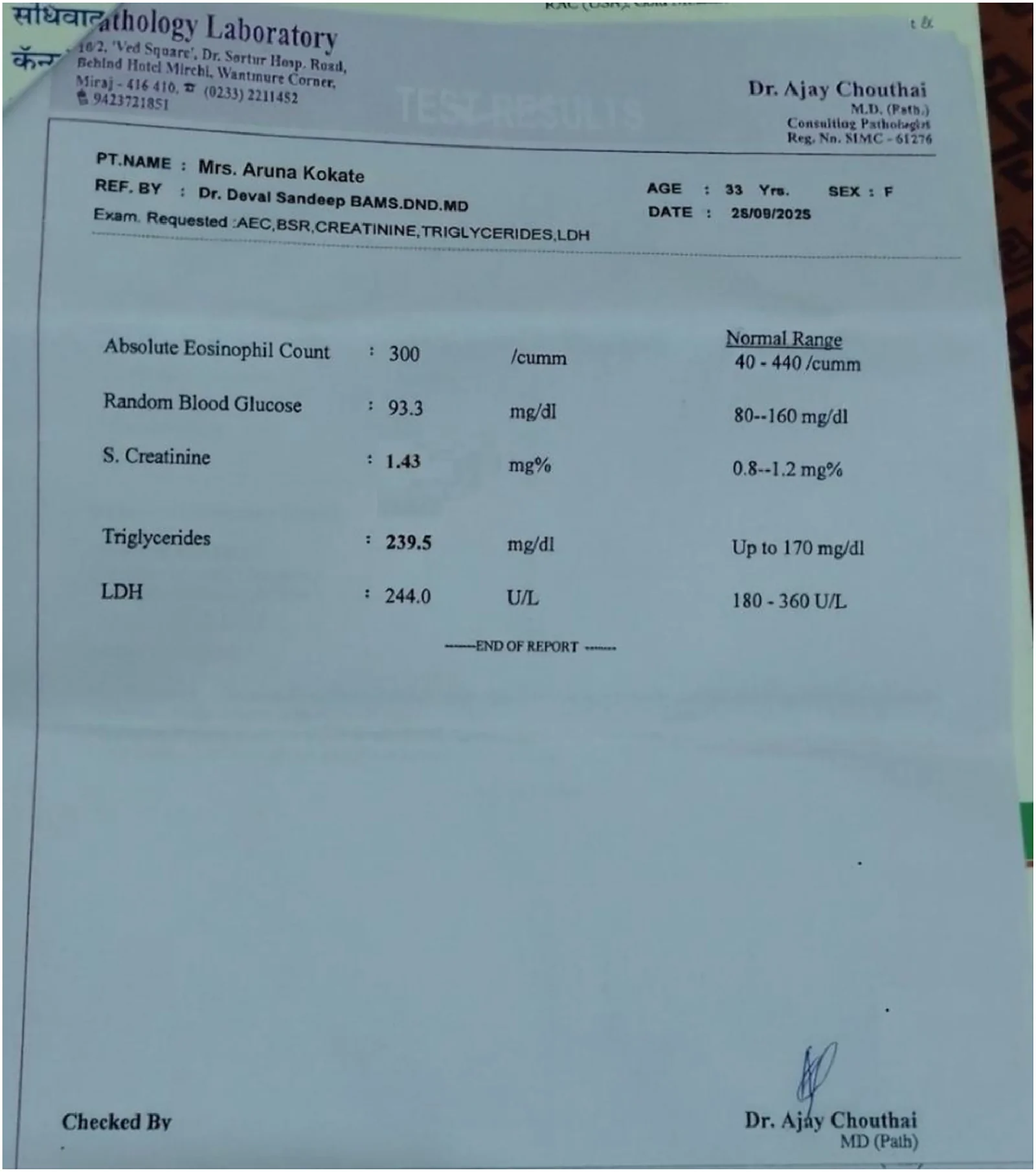
Pathology report of urine test.

## Limitations

The gas contents biomarkers in saliva gets evaporated over period of time. Hence the gas analysis shall be conducted immediately after taking saliva sample. Also the experimentation performance is affected by the warm-up time of sensors used. Hence the experimental setup should be turned ON and warmed up 15 min prior.

## Ethics statements

The research work involved the analysis of human saliva samples collected for experimental purposes. No human subjects were directly involved in the experimental procedures, and no intervention or medication treatment was performed on human participants on basis of this experimentation. A written consent was obtained from the participants before collecting the saliva sample.

## CRediT author statement

**Vicky Nalawade:** Sensor selection, System design and experimentation, Dataset creation, Initial paper drafting. **Vinayak K. Bairagi:** Validation, Editing and finalization of draft.

## Related research article

None.

## Declaration of competing interest

The authors declare that they have no known competing financial interests or personal relationships that could have appeared to influence the work reported in this paper.

## Data Availability

Data will be made available on request.
